# COVID‐19 outbreaks in aged‐care facilities in Australia

**DOI:** 10.1111/irv.12942

**Published:** 2021-12-05

**Authors:** Ashley Quigley, Haley Stone, Phi Yen Nguyen, Abrar Ahmad Chughtai, C. Raina MacIntyre

**Affiliations:** ^1^ The Kirby Institute UNSW Sydney Sydney New South Wales Australia; ^2^ School of Public Health and Community Medicine UNSW Sydney Sydney New South Wales Australia

**Keywords:** aged‐care, aged‐care facility, aged‐care worker, COVID‐19, epidemiology, public health

## Abstract

**Background:**

Aged‐care facilities (ACF’s) provide unique challenges when implementing infection control methods for respiratory outbreaks such as COVID‐19. Research on this highly vulnerable setting is lacking and there was no national reporting data of COVID‐19 cases in ACFs in Australia early in the pandemic. We aimed to estimate the burden of aged‐care worker (ACW) infections and outbreaks of COVID‐19 in Australian aged‐care.

**Methods:**

A line list of publicly available aged‐care related COVID‐19 reported cases from January 25 to June 10, 2020 was created and was enhanced by matching data extracted from media reports of aged‐care related COVID‐19 relevant outbreaks and reports. Rate ratios (RR) were used to predict risk of infection in ACW and aged‐care residents, and were calculated independently, by comparing overall cases to ACW and aged‐care residents' cases.

**Results:**

A total of 14 ACFs with COVID‐19 cases were recorded by June 2020 nationwide, with a high case fatality rate (CFR) of 50% (*n* = 34) and 100% (*n* = 3) seen in two ACFs. Analysis on the resident risk found that the COVID‐19 risk is 1.27 times higher (unadjusted RR 1.27 95% confidence interval [CI] 1.00 to1.61; *P* = 0.047) as compared with the risk of infection in the general population. In over 60% of cases identified in ACFs, the source of infection in the index case was unknown. A total of 28 deaths associated within ACFs were reported, accounting for 54.9% of total deaths in New South Wales and 26.9% of total deaths in Australia.

**Conclusions:**

This high‐risk population requires additional prevention and control measures, such as routine testing of all staff and patients regardless of symptoms. Prompt isolation and quarantine as soon as a case is confirmed within a facility is essential.

## 1 INTRODUCTION

As of October 29, 2021, over 246 million cases of COVID‐19 have been confirmed worldwide, causing more than 4.98 million deaths.^1^ Globally, high mortality rates have occurred in older patients,^1^, ^2^ and aged‐care has been a setting of serious outbreaks of COVID‐19.^3^, ^4^, ^5^ Case fatality rates among nursing home residents have been reported to be as high as 33%.^4^, ^6^, ^7^ The presence of comorbidities in older age patients are not only associated with increased risk of mortality from COVID‐19^8^ but the environment in aged‐care facilities (ACFs), where social distancing is difficult due to shared communal areas and close‐contact living, presents major barriers to infection control as well, contributing to the overall burden in these settings.^9^ A wide variation in the proportion of residents and staff being infected and in the spread of the disease between different ACFs has been reported.^4^, ^6^, ^7^

ACFs provide unique challenges when implementing infection control methods, especially considering respiratory outbreaks. The significant levels of close‐contact physical care^5^ combined with a less trained and prepared workforce^10^ who often face more difficulty accessing personal protective equipment (PPE), create difficulties in infection control measures. Not all aged‐care workers (ACWs) are required to be registered nurses with skilled health training and a very small percentage are qualified nurses.^11^ Studies on this high‐risk population is lacking, and there was previously no national reporting of data of ACF cases of COVID‐19. Despite the reporting of multiple COVID‐19 outbreaks in ACFs nationwide, the burden of COVID‐19 on Australian aged‐care has not been identified.^12^ We aimed to estimate national COVID‐19 outbreaks and cases in ACFs in Australia using open‐source data during the first wave of COVID‐19 in Australia.

## 2 METHODS

### 2.1 Data collection and data linkage

We collected publicly available data on Australian COVID‐19 patients and outbreaks reported by National and state/territory Governments and the media between January 25, 2020 and June 10, 2020. Cases were defined as people working or residing in ACFs with a diagnosis based on positive viral nucleic acid test results on respiratory swab samples. A line list of Australian aged‐care related COVID‐19 outbreaks in ACFs (where two or more infections between January 20, 2020 and June 10, 2020 were reported) was created. The line list also included aged‐care related cases in facilities where only a single case was identified. Data were obtained by searching the Australian Government Department of Health (DOH) Coronavirus (COVID‐19) current situation and case numbers daily update (supporting information) and individual state/territory health departments COVID‐19 daily update and press releases (supporting information) for officially reported individual cases in ACFs or those where an ACW was infected, and subsequent related outbreaks were made available to the public. Reporting practices changed markedly over the course of the pandemic and both National and Government COVID‐19 platforms for information sharing were monitored daily and recording of data was adapted accordingly. Data such as the name of the ACF, date, gender, age, location (state), and occupation (ACW) were recorded. For the purposes of this study, ACWs were defined as any employee who worked in an aged‐care setting during the COVID‐19 pandemic, regardless of clinical registration. Data were updated daily between January 25, 2020 and June 10, 2020, and no identifying information was recorded. Date of COVID‐19 confirmation by state pathology polymerase chain reaction (PCR) diagnosis was recorded as a proxy for onset date of symptoms.

To enhance the data set, data such as name of the ACF, date, gender, age, location (state), and occupation (ACW), where described, were extracted from media reports of aged‐care related COVID‐19 outbreaks and added to the line list (supporting information). A Google search was conducted between May 20, 2020 and June 10, 2020 using the following keywords: “aged care covid [state name]”, “nursing home covid [state name]”, and “aged care worker [state name]” where [state name] is repeated for Australia and all states and territories (New South Wales [NSW], South Australia [SA], Western Australia [WA], Tasmania [TAS], Victoria [VIC], Queensland [QLD], Northern Territory [NT], and Australian Capital Territory [ACT]). The first three pages of each Google search were reviewed.

ACF outbreaks were matched using date, location (state/territory and city), the name of the ACF, number of cases, patient age, number of deaths, and occupation as an ACW. This information was then matched between media reports found in the Google search and outbreak reports found on official government websites based on the matching criteria matrix in Table 1. A case is considered a high probability match if fulfilling at least one criterion from all Groups 1, 2, and 3; a medium probability match if fulfilling at least one criterion from Groups 2 and 3; and otherwise, a low probability match. Only cases with high probability matching were included for analysis. For the purposes of this study, ACFs were deidentified in reporting the results.

**TABLE 1:** High, medium, and low probability matching criteria for matching aged‐care related COVID‐19 cases

Group 1: Demographics	Group 2: Location	Group 3: Aged‐care details
Age	State	Name of aged‐care facility
Gender	City/Address	Name of aged‐care group + suburb
Occupation (ACW)		

*Note*: Criteria for high (at least one criterion from Groups 1, 2, and 3); medium (any criteria from Groups 2 and 3); and low (any criteria from Groups 1 and 3) are detailed.

Abbreviation: ACW, aged‐care workers.

### 2.2 Data analysis

Cumulative incidence rates of infections for ACWs were calculated using reported cases and the remaining total cases as the numerator and denominator data from the Australian Government, Institute of Health and Welfare Aged‐Care Data Services (AIHW GEN) (39). This was then repeated separately for aged‐care residents. The general population rates were calculated using notified national cases as the numerator and the Australian population as the denominator. Rate ratios (RRs) were calculated by comparing overall rates with those in ACW and aged‐care residents. Due to the absence of a complete data set where the age for each patient could be conclusively determined, the data were not age stratified. An epidemic curve was plotted to describe the distribution of aged‐care cases for both isolated cases and outbreaks, against Australia's nationwide epidemic curve.

Daily testing data for each state for all COVID‐19 tests performed were collected from individual state/territory health departments (supporting information) and plotted alongside aged‐care related COVID‐19 cases. Key dates relating to both federal government and state/territory control measures were overlaid to aid interpretation. To investigate the sources of infection among aged‐care, source acquisition data obtained from further media reports (supporting information) was collated and categorized based on whether the person who was the initial source of infection was identified and if so, had traveled overseas, contracted COVID‐19 in an ACF, or was a local case without any confirmed epidemiological linkage. The data were plotted to show distribution of sources. For each ACF identified, the number of staff in self quarantine, number of secondary related COVID‐19 infections, and the facility's policy of visitor restriction were recorded where possible, to determine the impact on the aged‐care workforce.^13^, ^14^, ^15^, ^16^, ^17^, ^18^

Analysis and reporting were based on the Strengthening the Reporting of Observational studies in Epidemiology (STROBE) guidelines for epidemiological studies.^19^ The data generated from this study were cleaned prior to analysis and presented using descriptive statistics after analysis with Stata IC version 16.1.^20^ A chi‐square test was used to calculate the RR.

## 3 RESULTS

### 3.1 Australian ACW COVID‐19 analysis

As of June 10, 2020, there had been 7276 cases in the total Australian population,^21^ equating to a crude cumulative incidence rate of 28.53/100,000. Data for COVID‐19 outbreaks in ACFs were obtained for 123 cases, in 14 ACFs; 6 of which were isolated cases, through analysis of open‐source data. Statistical analysis of the nationwide reported ACW COVID‐19 cases revealed that ACWs had a lower rate (unadjusted RR 0.50 95% confidence interval [CI] 0.39 to 0.65; *P* < 0.001) as compared with the general population at risk (Table 2). However, analysis of the cases in ACF residents risk found that the rate was 1.27 times higher (unadjusted RR 1.27 95% CI 1.00 to1.61; *P* = 0.047) as compared with the rate in the general population.

**TABLE 2:** Comparison of crude rates of COVID‐19 infections in workers (*n* = 55) and residents (*n* = 68) within aged‐care settings in Australia as of June 10, 2020

Category	Cumulative incidence (per 100,000)	Rate ratio	95% CI	*P* value
Australian population	28.53	—	—	—
ACW national^a^	0.2157	0.50	0.39 to 0.65	*P* < 0.001
Residents national^a^	0.2667	1.27	1.00 to 1.61	*P* = 0.047

Abbreviation: CI, confidence interval.

^a^ Statistically significant results.

**FIGURE 1:**
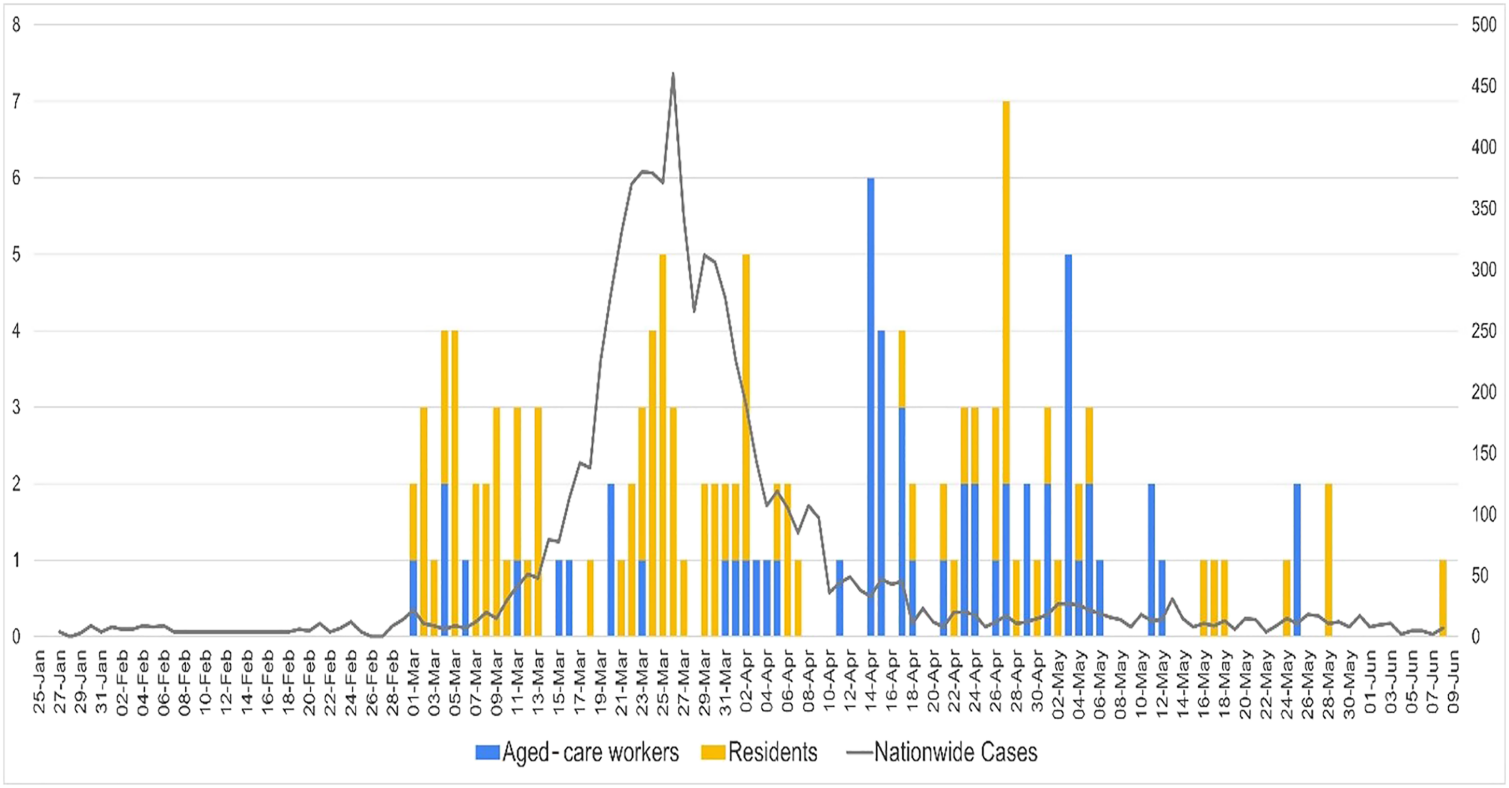
Epidemic curve of cases in ACFs from February 22, 2020 to May 29, 2020. The left *y*‐axis shows the daily number of aged‐care related COVID‐19 cases. The right *y*‐axis shows the total number of confirmed daily COVID‐19 cases nationwide. Date of COVID‐19 confirmation is used as a proxy for onset

Figure 1 summarizes the distribution of aged‐care related COVID‐19 cases and deaths reported by the states' departments of health and the media from January 25, 2020 to May 29, 2020, in a form of an epidemic curve. Although the overall COVID‐19 epidemic curve peaked on March 23–27, 2020, the epidemic curve for aged‐care related cases peaked within the prior 2‐week period, with relatively even distribution throughout the nationwide epidemic, whereas deaths lagged by 4 weeks. Most aged‐care related outbreaks that were identified occurred after April 2, 2020. Although the overall epidemic curve for Australia started to flatten in early April, aged‐care related cases persisted until May 28, 2020, with ACW infections predominant in this period.

### 3.2 COVID‐19 tests and ACW infections

**FIGURE 2:**
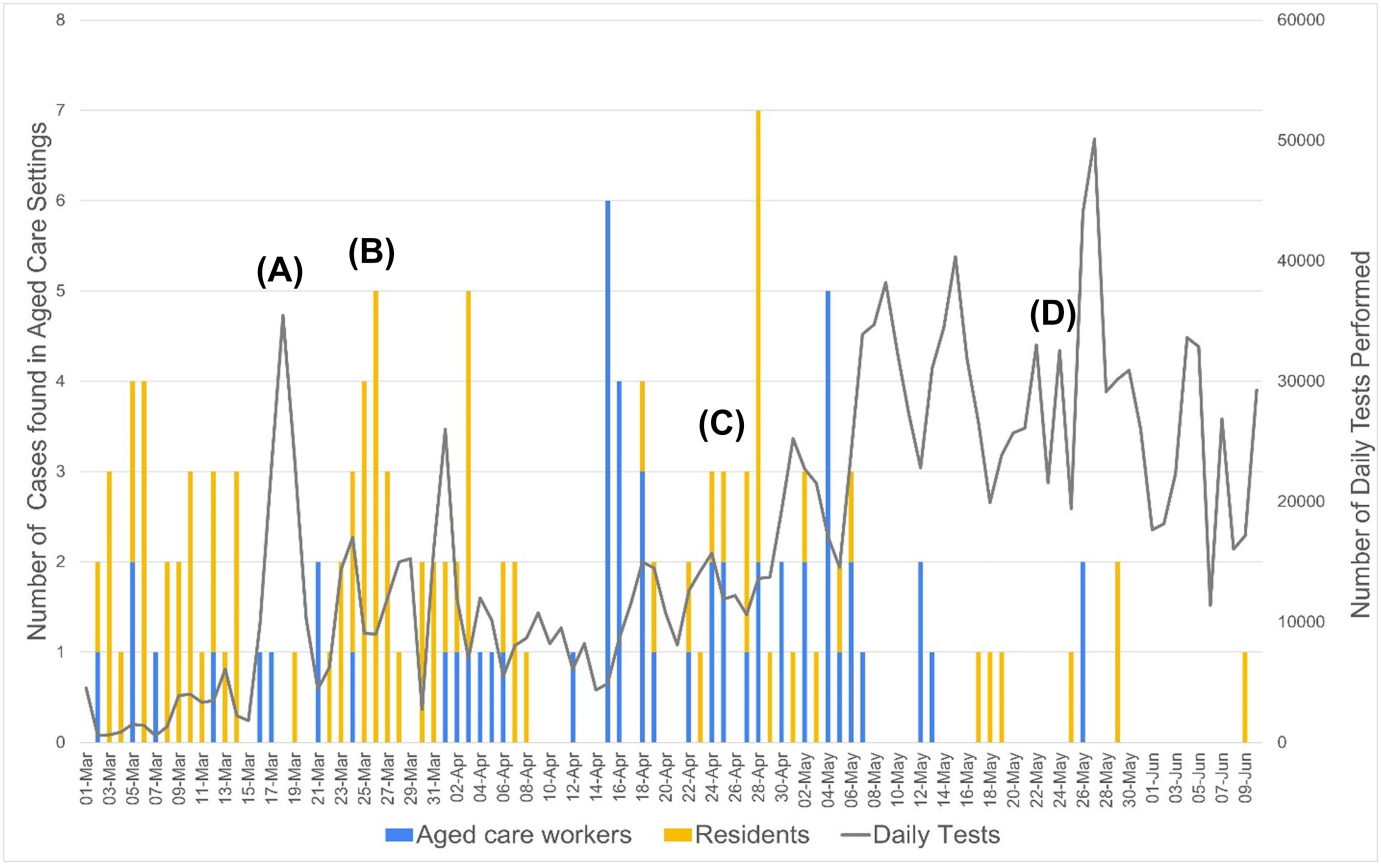
The left *y*‐axis shows the total number of confirmed aged‐care related COVID‐19 cases nationwide. The right *y*‐axis shows the number of daily COVID‐19 tests nationwide. Date of COVID‐19 confirmation is used as a proxy for onset. Expanded testing criteria (such as testing of asymptomatic high‐risk contacts in aged‐care facilities [ACFs]) will increase case detection and are indicated as follows: (A) March 17, QLD and ACT started regularly reporting testing data; (B) March 25, indicates a nationwide change in testing criteria to include people who did not recently travel from overseas; (C) May 1, indicates a change in reporting from people tested to number of tests performed in WA; (D) May 26/27, indicates a change in reporting from people tested to number of tests performed in NSW and VIC

Figure 2 shows the number of aged‐care related cases in relation to the number of daily COVID‐19 tests performed nationwide. Peaks in aged‐care reported cases also coincide with changes to the testing criteria implemented both nationwide and per state/territory, which occurred on March 25, 2020 to eliminate the requirement of recent travel in the criteria to test. The criteria then included those symptomatic with a fever. Since this national guideline within April, 2020, some individual states revised their policies based on the local epidemiology to include the testing of asymptomatic ACWs. Additionally, to increase awareness of the prevalence of COVID‐19 in the general population, NSW implemented a 2‐week enhanced testing blitz from April 27, 2020.^22^ Similarly, Victoria implemented an enhanced testing blitz from May 1, 2020,^23^ and South Australia implemented an enhanced testing blitz from May 16 to 30, 2020.^24^ March 25's nationwide expansion of testing criteria allowed all individuals exhibiting common symptoms of COVID‐19 to be tested, not just those exhibiting symptoms that had traveled overseas or close contacts of a case.

### 3.3 Sources of infection among ACWs

**FIGURE 3:**
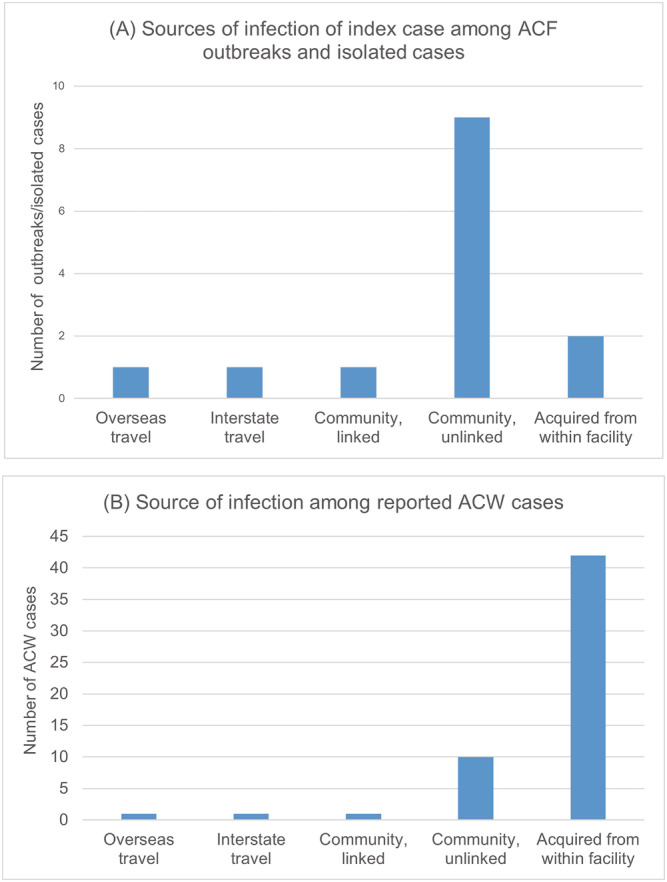
Frequency distribution of sources of COVID‐19 infection among ACF outbreaks and ACWs. ACF, aged‐care facilities; ACWs, aged‐care workers

Figure 3A,B summarizes the distribution of ACF and ACW cases from the ACFs with COVID‐19 cases, by source of infection. A total of 14 outbreaks and single cases in ACFs were identified as reporting COVID‐19 cases among staff and/or residents. Only three of the reported cases in ACFs were traced to an index case with a history of overseas travel (*n* = 1, 7.14%), interstate travel by flight (*n* = 1, 7.14%), or contact with a confirmed case (*n* = 1, 7.14%). In nine ACFs (64.3%), the source of infection in the index case was unknown (Figure 3A). Two ACF outbreaks were linked to an infected resident, which subsequently spread to an ACW. The source of infection in the residents was unknown.

A total of 55 ACW cases were reported from these facilities (Figure 3B). Out of 55 cases, 42 cases (76.4%) were diagnosed as part of contact tracing of the index case or reported within one incubation period (14 days) from the previous known case within the same facility. Ten cases (18.2%) acquired COVID‐19 without any history of overseas or interstate travel by flight or contact with a confirmed case.

Descriptive information for ACFs outbreaks is summarized in Table 3. A total of 28 deaths associated with COVID‐19 in ACFs were reported, accounting for 54.9% of total deaths in NSW and 26.9% of total deaths in Australia. All were aged‐care residents; no deaths among ACWs have been reported. Initial cases from Outbreak A were only formally tested positive after their deaths. One case tested negative, but the cause of death was later reclassified as COVID‐19 as it was deemed that complications from COVID‐19 contributed towards the patient's demise.^25^

**TABLE 3:** COVID‐19 affected ACFs identified between January 20 and June 10, 2020, in Australia

Date of notification	State	ACF outbreaks	Number of cases among ACWs	Number of cases among residents	Number of deaths among ACWs	Number of deaths among residents	CFR^a^	Description of index case	Source of infection for cases among ACWs	Facility restriction	Remark
04/03/2020–25/03/2020	NSW	A^a^	5	16	0	6	38%	ACW: Worked at facility while symptomatic before testing positive, with no overseas travel history.	Community, unlinked	Precautionary lockdown since notification.	Estimated 65% of regular staff went into self‐isolation. Residents were treated at Ryde Hospital, which was linked to the Ryde Hospital outbreak.
17/03/2020	WA	B^a^	1	0	0	0	0	ACW: Returning from Hawaii (US)—completed several shifts at facility before testing positive.	Overseas travel	No visitor entry from 20/03/2020 to 20/04/2020.	
18/03/2020	SA	C^a^	1	0	0	0	0	Allied health worker: Treated residents while asymptomatic on 16/03/2020.	Community, unlinked	No visitor entry from 18/03/2020 to 01/04/2020.	
22/03/2020; 06/04/2020–27/04/2020	NSW	D^a^	3	3	0	3	100%	Two staff tested positive in March. No information available on source of infection.	Community, unlinked	Precautionary lockdown during isolation period. No visitor entry from 18/03/2020, with progressive easing of restriction for compassionate visits since mid‐April.	Latest death was revised as COVID‐19 related despite two negative swabs.
23/03/2020–25/03/2020	NSW	E^a^	0	2	0	0	0	Resident	Within facility	Precautionary lockdown during isolation period. No information on visitor restriction.	
03/2020; 03/04/2020–30/04/2020	VIC	F^a^	2	3	0	0	0	ACWs: First case was diagnosed in early March and second case 3 weeks later; 3 later cases were found as part of contact tracing of the second case.	Community, unlinked	Precautionary lockdown from 01/05/2020. No information on visitor restriction.	
02/04/2020–07/04/2020	NSW	G^a^	2	0	0	0	0	ACW: Last worked at facility on 26/03/2020 and tested positive on 02/04/2020.	Community, unlinked	Precautionary lockdown from first notification to 14/04/2020. No visitor entry from 30/03/2020 to 25/05/2020.	
04/04/2020	NSW	H^a^	1	0	0	0	0	No information available.	Community, unlinked	No visitor entry from 30/03/2020 to 31/05/2020	Infected staff worked at both facilities.
05/04/2020	NSW	I^a^	1	0	0	0	0	No information available.	Community, unlinked	No visitor entry from 28/03/2020 to 24/04/2020	
16/04/2020–13/05/2020	NSW	J^a^	34	37	0	17	51%	ACW: Worked at facility for 6 consecutive days while asymptomatic before testing positive.	Community, unlinked	Precautionary lockdown from mid‐April. No visitor entry from start of outbreak until 15/06/2020.	During the first week of the outbreak, up to 50% of regular staff went into self‐isolation.
05/05/2020	VIC	K^a^	1	0	0	0	0	ACW: Close contact of a worker at Cedar Meats abattoir, Victoria's largest outbreak. Last worked at facility on 26/04/2020.	Community, linked to confirmed case.	No visitor entry since 18/3/2020.	
05/05/2020	VIC	L^a^	1	0	0	0	0	ACW: Asymptomatic, tested as part of Victoria's testing blitz.	Community, unlinked	No visitor entry from 05/05/2020 to 11/05/2020.	
14/05/2020	QLD	M^a^	1	0	0	0	0	ACW: Traveled to Brisbane via flight; worked at facility while symptomatic until tested positive (14/05/2020).	Interstate travel (via flight).	Precautionary lockdown from first notification until 01/06/2020. No visitor entry from 01/06/2020 to 05/06/2020.	
19/05/2020–27/05/2020	VIC	N^a^	2	1	0	0	0	Resident: unlinked to two later cases which were ACWs.	Within facility	Precautionary lockdown from 19/05/2020.	

Abbreviations: ACF, aged‐care facilities; ACT, Australian Capital Territory; ACWs, aged‐care workers; CFR, case fatality rate; NSW, New South Wales; NT, Northern Territory; QLD, Queensland; SA, South Australia; TAS, Tasmania; VIC, Victoria; WA, Western Australia.

^a^ Sources may be obtained from the supporting information.

Most ACFs had implemented a ban on external visitors prior to their first case, allowing only case‐by‐case visits on compassionate grounds. All ACFs outbreaks with at least five cases (staff and residents combined) occurred in facilities that had not implemented such a ban until the first case was reported. All facilities executed precautionary lockdown upon notification of the first case, for a minimum period of 14 days, followed by restricted entry policies. The length of restricted entry varies from facility to facility, depending on state and facility policy.

## 4 DISCUSSION

We found that the risk of COVID‐19 in residents of aged‐care settings is 1.27 times higher than the general population. This would be a crude, minimal estimate, because testing policy was restricted in the initial months and it was not a routine to test asymptomatic people, and data were not age stratified. Studies of long‐term care facility outbreaks in the United States show that attack rates are high and asymptomatic or pre‐symptomatic infection is common.^4^, ^6^ Therefore, if asymptomatic staff and residents are not tested during an outbreak, the true incidence of COVID‐19 will be underestimated. The peaks seen in ACW infections nationwide occurred after the expansion of testing by national state territories during April 2020^22^, ^23^, ^24^ where expansion of testing was implemented to include those with all cold‐like symptoms (but not asymptomatic people), reinforcing the likely under‐ascertainment of ACW cases, contributing to the idea that testing in aged‐care nationally needs increasing. Expanding testing allows for the quick identification of cases in vulnerable populations such as the elderly and the aged‐care workforce.

As symptom‐based screening of residents might fail to identify all COVID‐19 cases infections,^6^ minimizing exposure of the vulnerable high‐risk residents in future outbreaks is needed. The analysis of ACW infections showed there was no associated risk of COVID‐19 in ACWs, who in fact had a lower risk than the general community. This may be due to under‐ascertainment and under‐testing in this group, who are more likely to be asymptomatic because of younger age. It may also be possible that the rapid response in aged‐care settings by health authorities during this pandemic limited the spread. It was also reported in some outbreaks that ACWs refused to return to work, so exposure may have been reduced by work absenteeism.

Our analysis included isolated cases of COVID‐19 within ACFs, as the use of single cases will likely reduce the underestimation of outbreaks in this study period. A CFR of 100% (3 of 3) was seen in the outbreak in Outbreak D, whereas a CFR of 51% was seen in Outbreak J, where 17 deaths occurred and 37 residents were infected. The source of infection for this outbreak was traced back to a single ACW, who worked while symptomatic,^18^ again highlighting the need for increased testing and infection control in aged‐care settings. In addition, deaths in ACFs accounted for a high proportion of all COVID‐19 related deaths in Australia, especially in the state of NSW.^26^ The data from the Victorian second wave suggest ACW are at higher risk than healthcare workers.^27^

Visitors and staff are the usual vector of introduction of infection into a facility, whereas residents tend not to move in and out of the facility. This study found that in over 60% of cases, the source of infection in the index case was unknown, highlighting the risk of outbreaks being seeded in aged‐care facilities. Over 70% of the COVID‐19 infections in ACWs were diagnosed as part of contact tracing of the index case or reported within one incubation period (14 days) from the previous known case within the same facility. Thus, an up‐to‐date registration or enumeration of the aged‐care workforce, not just on a national level, but for each state or territory, is vital. ACWs can work either in full‐time or casual positions, and one ACW may work at more than one facility at the same time, complicating the contact tracing process and increasing the risk of transmission between multiple facilities. Facilities with a confirmed COVID‐19 case should rapidly implement the use of recommended PPE when caring for residents^4^ as per the guidelines provided by the Australian DOH^28^; PPE should be worn whenever someone with a confirmed OR suspected case of COVID‐19 is being cared for.^28^ A combination of increased surveillance, infection control, and mitigation efforts are essential to decreasing the transmission of COVID‐19, especially among aged‐care facilities.

A potential limitation of the risk analyses conducted for this study were based on open‐sourced data for ACF cases, which may vary depending on each state's individual data publishing policies. This is likely to lead to underestimation of cases in ACF. We also used media reports, which have not been verified. In most cases, however, there were multiple media reports about each outbreak, often with a quote from health officials. However due to the absence of a complete data set where the age for each patient could be conclusively determined, the data were not age stratified. There is also a potential effect of testing rates on the identification of COVID‐19 cases. We accounted for this by representing the daily testing rates in conjunction with the daily ACF and ACW infections reported. A large proportion of ACW and ACF infections had an unknown source, and we were unable to obtain data on source of infection, nor verify reported sources. This highlights the need for proper and detailed outbreak investigation and contact tracing during aged‐care outbreaks. This study did not investigate the second wave in Melbourne. This study may not be generalizable to other countries or settings, with different COVID‐19 epidemiology, PPE policies, and health systems.

## 5 CONCLUSION

ACFs are a high‐risk setting for COVID‐19 outbreaks. Fourteen outbreaks of COVID‐19 occurred in Australia by June 2020, with a high CFR. Commonly, the source of initial infection was unknown. Interventions for preventing COVID‐19 transmission needs to consider the high possibility of asymptomatic transmission, testing of all residents and staff during an outbreak, and prompt isolation and quarantine as soon as a case is confirmed within a facility. This high‐risk population requires additional prevention measures, which may be tailored to the level of community transmission. We also recommend registration or enumeration of the aged‐care workforce for each state or territory.

## AUTHOR CONTRIBUTIONS

**Ashley Quigley:** Writing‐original draft; project administration; conceptualization; methodology; formal analysis. **Haley Stone:** Methodology; formal analysis. **Phi Yen Nguyen:** Methodology; formal analysis. **Abrar Chughtai:** Supervision; writing‐review and editing. **C. Raina MacIntyre:** Conceptualization; supervision; writing‐review and editing.

6

### PEER REVIEW

The peer review history for this article is available at https://publons.com/publon/10.1111/irv.12942.

## Supporting information


**Data S1.** Supporting Information

## Data Availability

Data sharing is not applicable to this article as no datasets were generated or analyzed during the current study.
