# Supplementary material for: COVID‐19 outbreaks in aged‐care facilities in Australia
Source: Influenza Other Respir Viruses. 2021 Dec 5;16(3):429–37. doi: 10.1111/irv.12942 (PMC8983895; doi:10.1111/irv.12942)
Supplement: Supplementary file 1 — Data S1. Supporting Information [file IRV-16-429-s001.docx]

**Supplementary File:**

**National and Government COVID-19 sources:**

1. (DOH) Coronavirus (COVID-19) current situation and case numbers daily update^1^.
2. Individual state/territory Health Departments COVID-19 daily update and press releases ^2–11^.

**Sources used for dataset enhancement:**

1. To enhance the data set, data such as name of the Aged-care facility, date, gender, age, location (state), and occupation (ACW), where described, were extracted from media reports of aged-care related COVID-19 cases and added to the line list ^12,13,22–24,14–21^.

**Daily Testing Data:**

1. Daily testing data for each state for all COVID-19 tests performed was collected from individual state/territory Health Departments ^5,8,11,25–29^

**Source Acquisition:**

1. To investigate the sources of infection amongst aged-care, source acquisition data obtained from further media reports ^12,13,22–24,26,14–21^

**Table 3:** COVID-19 aged-care related clusters with 2 or more infections between January 20^th^, 2020 and June 10^th^, 2020 in Australia.

| **Date of notification** | **State** | **Cluster** | **Number of cases among ACW’s** | **Number of cases among residents** | **Number of deaths among ACW’s** | **Number of deaths among residents** | **CFR*** | **Description of index case** | **Source of infection for cases among ACW’s** | **Facility Restriction** | **Remark** |
| --- | --- | --- | --- | --- | --- | --- | --- | --- | --- | --- | --- |
| 04/03/2020-25/03/2020 | NSW | A ^12,13^ | 5 | 16 | 0 | 6 | 38% | ACW: Worked at facility while symptomatic before testing positive, with no overseas travel history. | Community, unlinked | Precautionary lockdown since notification. | Estimated 65% of regular staff went into self-isolation.  Residents were treated at Ryde Hospital, which was linked to the Ryde Hospital outbreak. |
| 17/03/2020 | WA | B ^14,15^ | 1 | 0 | 0 | 0 | 0 | ACW: Returning from Hawaii (US) - completed several shifts at facility before testing positive. | Overseas travel | No visitor entry from 20/03/2020 - 20/04/2020. |  |
| 18/03/2020 | SA | C ^16^ | 1 | 0 | 0 | 0 | 0 | Allied health worker: Treated residents whilst asymptomatic on 16/03/2020. | Community, unlinked | No visitor entry from 18/03/2020-01/04/2020. |  |
| 22/03/2020; 06/04/2020-27/04/2020 | NSW | D ^17,30^ | 3 | 3 | 0 | 3 | 100% | Two staff tested positive in March. No information available on source of infection. | Community, unlinked | Precautionary lockdown during isolation period. No visitor entry from 18/03/2020, with progressive easing of restriction for compassionate visits since mid-April. | Latest death was revised as COVID-19 related despite two negative swabs. |
| 23/03/2020-25/03/2020 | NSW | E ^30^ | 0 | 2 | 0 | 0 | 0 | Resident | Within facility | Precautionary lockdown during isolation period. No information on visitor restriction. |  |
| 03/2020; 03/04/2020-30/04/2020 | VIC | F ^18^ | 2 | 3 | 0 | 0 | 0 | ACW’s: First case was diagnosed in early March and second case 3 weeks later; 3 later cases were found as part of contact tracing of the second case. | Community, unlinked | Precautionary lockdown from 01/05/2020.  No information on visitor restriction. |  |
| 02/04/2020- 07/04/2020 | NSW | G ^19^ | 2 | 0 | 0 | 0 | 0 | ACW: Last worked at facility on 26/03/2020 and tested positive on 02/04/2020. | Community, unlinked | Precautionary lockdown from first notification to 14/04/2020. No visitor entry from 30/03/2020-25/05/2020. |  |
| 04/04/2020 | NSW | H ^30^ | 1 | 0 | 0 | 0 | 0 | No information available. | Community, unlinked | No visitor entry from 30/03/2020-31/05/2020 | Infected staff worked at both facilities. |
| 05/04/2020 | NSW | I ^20^ | 1 | 0 | 0 | 0 | 0 | No information available. | Community, unlinked | No visitor entry from 28/03/2020-24/04/2020 |  |
| 16/04/2020-13/05/2020 | NSW | J ^21^ | 34 | 37 | 0 | 17 | 51% | ACW: Worked at facility for 6 consecutive days while asymptomatic before testing positive. | Community, unlinked | Precautionary lockdown from mid-April.  No visitor entry from start of outbreak until 15/06/2020. | During the first week of the outbreak, up to 50% of regular staff went into self-isolation. |
| 05/05/2020 | VIC | K ^22^ | 1 | 0 | 0 | 0 | 0 | ACW: Close contact of a worker at Cedar Meats abattoir, Victoria's largest outbreak. Last worked at facility on 26/04/2020. | Community, linked to confirmed case. | No visitor entry since 18/3/2020. |  |
| 05/05/2020 | VIC | L ^22^ | 1 | 0 | 0 | 0 | 0 | ACW: Asymptomatic, tested as part of Victoria's testing blitz. | Community, unlinked | No visitor entry from 05/05/2020-11/05/2020. |  |
| 14/05/2020 | QLD | M ^23^ | 1 | 0 | 0 | 0 | 0 | ACW: Travelled to Brisbane via flight; worked at facility while symptomatic until tested positive (14/05/2020). | Interstate travel (via flight). | Precautionary lockdown from first notification until 01/06/2020. No visitor entry from 01/06/2020-05/06/2020. |  |
| 19/05/2020-27/05/2020 | VIC | N ^24^ | 2 | 1 | 0 | 0 | 0 | Resident: unlinked to two later cases which were ACW’s. | Within facility | Precautionary lockdown from 19/05/2020. |  |

**Reference List**

1. Coronavirus (COVID-19) current situation and case numbers. Australian Ministry Department of Health (DOH). https://www.health.gov.au/news/health-alerts/novel-coronavirus-2019-ncov-health-alert/coronavirus-covid-19-current-situation-and-case-numbers. Published 2020. Accessed September 1, 2020.

2. NSW Department of Health. Media releases from NSW Health. https://www.health.nsw.gov.au/news/pages/2020-nsw-health.aspx. Published 2020. Accessed July 10, 2020.

3. Queensland Government. QLD Department Of Health Media Releases. https://www.health.qld.gov.au/news-events/doh-media-releases. Published 2020. Accessed July 10, 2020.

4. Tasmanian Government Department of Health. TAS News Releases. https://www.dhhs.tas.gov.au/news. Published 2020. Accessed July 10, 2020.

5. Victoria State Government; Health and Human Services. Media hub - coronavirus disease (COVID-19). https://www.dhhs.vic.gov.au/media-hub-coronavirus-disease-covid-19. Published 2020. Accessed July 10, 2020.

6. Government of South Australia. SA Media Releases. https://www.sahealth.sa.gov.au/wps/wcm/connect/public+content/sa+health+internet/about+us/news+and+media/all+media+releases?mr-sort=date-desc&mr-pg=1. Published 2020. Accessed July 10, 2020.

7. Government of Western Australia Department of Health. Latest News: Media Releases. https://ww2.health.wa.gov.au/News/Media-releases-listing-page. Published 2020. Accessed July 10, 2020.

8. Northern Territory Government. Coronavirus (COVID-19). https://coronavirus.nt.gov.au/updates. Published 2020. Accessed July 10, 2020.

9. ACT Government. ACT Government: Covid-19. https://www.covid19.act.gov.au/. Published 2020. Accessed July 10, 2020.

10. Victoria State Government. Media releases. https://www2.health.vic.gov.au/about/media-centre/mediareleases. Published 2020. Accessed July 10, 2020.

11. Government of Tasmania. Coronavirus Disease (COVID-19) - Cases and Testing Updates. Media Releases. https://www.coronavirus.tas.gov.au/media-releases. Published 2020. Accessed May 18, 2020.

12. Daoud E. Coronavirus in Australia : Death toll rises as aged care resident from Dorothy Henderson Lodge dies from COVID-19. https://7news.com.au/lifestyle/health-wellbeing/coronavirus-in-sydney-another-aged-care-resident-from-the-dorothy-henderson-lodge-dies-from-covid-19-c-959773. Published 2020. Accessed June 25, 2020.

13. Wahlquist C. “Stranger in a hazmat suit”: families of Dorothy Henderson Lodge residents try to stay positive. The Guardian; Australian Edition. https://www.theguardian.com/australia-news/2020/apr/05/stranger-in-a-hazmat-suit-families-of-dorothy-henderson-lodge-residents-try-to-stay-positive. Published 2020. Accessed June 25, 2020.

14. May R Le. Infected worker sparks aged care lockdown. 7 News. https://7news.com.au/news/health/wa-premier-rejects-state-border-close-call-c-749114. Published 2020. Accessed May 20, 2020.

15. Coronavirus (COVID-19) Update Visiting Restrictions Apply Facility Lockdown To Follow. Aegis Aged Care Group. https://aegiscare.com.au/downloads/pdf/home/aegis_coronavirus.pdf. Published 2020. Accessed June 25, 2020.

16. COVID 19 Statement. St Louis Aged Care. https://www.stlouisagedcare.com.au/blog/covid-19-statement/. Published 2020. Accessed June 25, 2020.

17. Information on COVID-19 - Opal Aged Care Visitor arrangement update: Tuesday 23 June 2020. Opal Aged Care. https://www.opalagedcare.com.au/information-on-covid-19. Published 2020. Accessed June 25, 2020.

18. Taylor J. Victorian coronavirus cluster in aged care home under investigation News is under threat …. The Guardian; Australia Edition. https://www.theguardian.com/world/2020/may/01/victorian-coronavirus-cluster-in-aged-care-home-under-investigation. Published 2020. Accessed May 24, 2020.

19. Anglican Care Media Statement. Second employee of Storm Village, Taree tests positive with COVID-19. Great Lakes Advocate. https://www.greatlakesadvocate.com.au/story/6716779/another-storm-staff-member-tests-positive-with-covid-19/. Published 2020. Accessed June 25, 2020.

20. Dvorak S. Media Report: First confirmed COVID-19 case in Bupa Aged Care Australia – employee tests positive. Bupa Health & Care. https://media.bupa.com.au/first-confirmed-covid-19-case-in-bupa-aged-care-australia--employee-tests-positive/. Published 2020. Accessed May 24, 2020.

21. Anglicare’s Response to Coronavirus ( COVID-19 ). anglicare.org.au/about-us/media-releases/coronavirus-covid-19%0ACommunity. Published 2020. Accessed June 25, 2020.

22. Sakkal P. Nursing home residents tested after staff members’ positive results. The Age. https://www.theage.com.au/national/victoria/bacchus-marsh-nursing-home-in-lockdown-after-staff-member-tests-positive-20200506-p54qdv.html. Published 2020. Accessed May 24, 2020.

23. Jarrett V, Smith L. UPDATE : CQUni says sick nurse visited library numerous times. Western Star News. https://www.westernstarnews.com.au/news/revealed-where-rockys-latest-covid-19-case-is-loca/4016212/. Published 2020. Accessed June 25, 2020.

24. Chapman A. Lynden Aged Care in Camberwell forced to isolate staff and residents after workers catch coronavirus. 7 News. https://7news.com.au/lifestyle/health-wellbeing/lynden-aged-care-in-camberwell-forced-to-isolate-staff-and-residents-after-workers-catch-coronavirus-c-1061873. Published 2020. Accessed May 30, 2020.

25. NSW Government. NSW COVID-19 cases data. Data.NSW. https://data.nsw.gov.au/nsw-covid-19-data/cases. Published 2020. Accessed June 20, 2020.

26. Coronavirus (COVID-19) current situation and case numbers. Australian Government Department of Health. https://www.health.gov.au/news/health-alerts/novel-coronavirus-2019-ncov-health-alert/coronavirus-covid-19-current-situation-and-case-numbers%0Ahttp://files/358/coronavirus-covid-19-current-situation-and-case-numbers.html. Published 2020. Accessed November 10, 2020.

27. Government of South Australia. Dashboard and daily update. SA.GOV.AU: COVID-19. https://www.covid-19.sa.gov.au/home/dashboard. Published 2020. Accessed July 10, 2020.

28. Queensland Government. Queensland COVID-19 statistics. https://www.qld.gov.au/health/conditions/health-alerts/coronavirus-covid-19/current-status/statistics. Published 2020. Accessed July 10, 2020.

29. Government of Western Australia Department of Health. Coronavirus COVID-19 in Western Australia. https://experience.arcgis.com/experience/359bca83a1264e3fb8d3b6f0a028d768. Accessed June 20, 2020.

30. NSW Department of Health. COVID-19 (Coronavirus) statistics. https://www.health.nsw.gov.au/news/Pages/20200411_00.aspx. Published 2020. Accessed July 10, 2020.

| **Date of notification** | **State** | **Cluster** | **Number of cases among ACW’s** | **Number of cases among residents** | **Number of deaths among ACW’s** | **Number of deaths among residents** | **CFR*** | **Description of index case** | **Source of infection for cases among ACW’s** | **Facility Restriction** | **Remark** |
| --- | --- | --- | --- | --- | --- | --- | --- | --- | --- | --- | --- |
| 04/03/2020-25/03/2020 | NSW | A ^12,13^ | 5 | 16 | 0 | 6 | 38% | ACW: Worked at facility while symptomatic before testing positive, with no overseas travel history. | Community, unlinked | Precautionary lockdown since notification. | Estimated 65% of regular staff went into self-isolation.  Residents were treated at Ryde Hospital, which was linked to the Ryde Hospital outbreak. |
| 17/03/2020 | WA | B ^14,15^ | 1 | 0 | 0 | 0 | 0 | ACW: Returning from Hawaii (US) - completed several shifts at facility before testing positive. | Overseas travel | No visitor entry from 20/03/2020 - 20/04/2020. |  |
| 18/03/2020 | SA | C ^16^ | 1 | 0 | 0 | 0 | 0 | Allied health worker: Treated residents whilst asymptomatic on 16/03/2020. | Community, unlinked | No visitor entry from 18/03/2020-01/04/2020. |  |
| 22/03/2020; 06/04/2020-27/04/2020 | NSW | D ^17,30^ | 3 | 3 | 0 | 3 | 100% | Two staff tested positive in March. No information available on source of infection. | Community, unlinked | Precautionary lockdown during isolation period. No visitor entry from 18/03/2020, with progressive easing of restriction for compassionate visits since mid-April. | Latest death was revised as COVID-19 related despite two negative swabs. |
| 23/03/2020-25/03/2020 | NSW | E ^30^ | 0 | 2 | 0 | 0 | 0 | Resident | Within facility | Precautionary lockdown during isolation period. No information on visitor restriction. |  |
| 03/2020; 03/04/2020-30/04/2020 | VIC | F ^18^ | 2 | 3 | 0 | 0 | 0 | ACW’s: First case was diagnosed in early March and second case 3 weeks later; 3 later cases were found as part of contact tracing of the second case. | Community, unlinked | Precautionary lockdown from 01/05/2020.  No information on visitor restriction. |  |
| 02/04/2020- 07/04/2020 | NSW | G ^19^ | 2 | 0 | 0 | 0 | 0 | ACW: Last worked at facility on 26/03/2020 and tested positive on 02/04/2020. | Community, unlinked | Precautionary lockdown from first notification to 14/04/2020. No visitor entry from 30/03/2020-25/05/2020. |  |
| 04/04/2020 | NSW | H ^30^ | 1 | 0 | 0 | 0 | 0 | No information available. | Community, unlinked | No visitor entry from 30/03/2020-31/05/2020 | Infected staff worked at both facilities. |
| 05/04/2020 | NSW | I ^20^ | 1 | 0 | 0 | 0 | 0 | No information available. | Community, unlinked | No visitor entry from 28/03/2020-24/04/2020 |  |
| 16/04/2020-13/05/2020 | NSW | J ^21^ | 34 | 37 | 0 | 17 | 51% | ACW: Worked at facility for 6 consecutive days while asymptomatic before testing positive. | Community, unlinked | Precautionary lockdown from mid-April.  No visitor entry from start of outbreak until 15/06/2020. | During the first week of the outbreak, up to 50% of regular staff went into self-isolation. |
| 05/05/2020 | VIC | K ^22^ | 1 | 0 | 0 | 0 | 0 | ACW: Close contact of a worker at Cedar Meats abattoir, Victoria's largest outbreak. Last worked at facility on 26/04/2020. | Community, linked to confirmed case. | No visitor entry since 18/3/2020. |  |
| 05/05/2020 | VIC | L ^22^ | 1 | 0 | 0 | 0 | 0 | ACW: Asymptomatic, tested as part of Victoria's testing blitz. | Community, unlinked | No visitor entry from 05/05/2020-11/05/2020. |  |
| 14/05/2020 | QLD | M ^23^ | 1 | 0 | 0 | 0 | 0 | ACW: Travelled to Brisbane via flight; worked at facility while symptomatic until tested positive (14/05/2020). | Interstate travel (via flight). | Precautionary lockdown from first notification until 01/06/2020. No visitor entry from 01/06/2020-05/06/2020. |  |
| 19/05/2020-27/05/2020 | VIC | N ^24^ | 2 | 1 | 0 | 0 | 0 | Resident: unlinked to two later cases which were ACW’s. | Within facility | Precautionary lockdown from 19/05/2020. |  |
